# Importance of the pyrolysis for microstructure and superconducting properties of CSD-grown GdBa_2_Cu_3_O_7−x_-HfO_2_ nanocomposite films by the ex-situ approach

**DOI:** 10.1038/s41598-020-75587-4

**Published:** 2020-11-10

**Authors:** Pablo Cayado, Hannes Rijckaert, Els Bruneel, Manuela Erbe, Jens Hänisch, Isabel Van Driessche, Bernhard Holzapfel

**Affiliations:** 1 Karlsruhe Institute of Technology (KIT), Institute for Technical Physics (ITEP), Hermann-von-Helmholtz-Platz 1, 76344 Eggenstein-Leopoldshafen, Germany; 2 Department of Chemistry, Ghent University, SCRiPTS, Krijgslaan, 281-S3, 9000 Ghent, Belgium

**Keywords:** Materials science, Nanoscience and technology

## Abstract

For the first time, GdBa_2_Cu_3_O_7−*x*_ nanocomposites were prepared by chemical solution deposition following the ex-situ approach. In particular, ~ 220 nm GdBa_2_Cu_3_O_7−*x*_-HfO_2_ (GdBCO-HfO_2_) nanocomposite films were fabricated starting from a colloidal solution of 5 mol% HfO_2_ nanoparticles. Hereby, one of the main challenges is to avoid the accumulation of the nanoparticles at the substrate interface during the pyrolysis, which would later prevent the epitaxial nucleation of the GdBCO grains. Therefore, the effect of pyrolysis processing parameters such as heating ramp and temperature on the homogeneity of the nanoparticle distribution has been investigated. By increasing the heating ramp to 300 °C/h and decreasing the final temperature to 300 °C, a more homogenous nanoparticle distribution was achieved. This translates into improved superconducting properties of the grown films reaching critical temperatures (*T*_c_) of 94.5 K and self-field critical current densities (${J}_{\mathrm{c}}^{\mathrm{sf}}$) at 77 K of 2.1 MA/cm^2^ with respect to films pyrolyzed at higher temperatures or lower heating ramps.

## Introduction

The development of coated conductors (CCs) is one of the major research issues in applied superconductivity^1–5^. CCs are flexible tapes based on a metallic substrate and a layered architecture on top, where the key component is a superconducting film made of *RE*Ba_2_Cu_3_O_7−δ_ (*RE*BCO, *RE* rare earth). These compounds have high critical temperatures (*T*_c_) and can carry high currents in applied magnetic fields, which makes them adequate materials for a number of power applications such as motors or generators.

Yet, the use of CCs in such applications is still not widespread. One of the main reasons is that *RE*BCO films do not fully meet the operational requirements of the devices. Therefore, the improvement of the superconducting properties of these films, especially at high magnetic fields, is an important topic.

One possibility to improve the performance of CCs is to substitute the well-known and commonly used YBCO with alternative *RE*BCO compounds, since they often display improved superconducting properties^6–10^. One of them is GdBCO, with *T*_c_ values up to 95 K in bulk samples, single crystals and thin films^6,11,12^. In particular, the GdBCO thin films were often fabricated by in-situ techniques, where the expression in situ is related to the crystallization process of the *RE*BCO phase: metal–organic chemical vapour deposition (MOCVD), pulsed laser deposition (PLD) or sputtering^13–17^. However, chemical solution deposition (CSD) appears to be an attractive alternative to the previous techniques. It is a scalable, versatile and low-cost technique that follows a chemical route for the growth of the films^18–22^ and is thus suitable for the preparation of CCs. Very popular and thoroughly studied is the TFA-MOD route, which is a particular CSD technique leading to excellent superconducting properties in GdBCO films^12,23–31^.

Another strategy offering significant performance enhancements is the introduction of secondary phases in the films. The preparation of nanocomposites through the incorporation of nanoparticles (NPs) by CSD has been studied attentively for several years, whereby most of the efforts were devoted to research on the so-called “in-situ” approach. In this approach, the NPs segregate spontaneously in the *RE*BCO matrix during the thermal processes. The resulting films present excellent critical current densities at 77 K even exceeding 7 MA/cm^2^ as well as improved in-field performances^9,32–35^. However, this approach has one major disadvantage for further improvements of the superconducting properties: the poor control of the NP size and distribution in the matrix. To overcome this issue, the “ex-situ” approach was recently developed. For that, a colloidal dispersion of preformed NPs is mixed with the *RE*BCO precursor solution and deposited on a substrate. The NPs are embedded within the matrix in the subsequent pyrolysis and growth process. This approach has the potential to overcome the limitations of the in-situ approach because preformed NPs can be tailored with specific sizes and shapes. Recent publications showed indeed that films prepared with this technique have promising perspectives to compete with and even surpass the in-situ appoach^36–41^.

In this work, we tried to combine the benefits of an alternative *RE*BCO compound, here GdBCO, with the ex-situ approach. Although this paper presents the first results on pre-formed particles with a *RE*BCO compound other than YBCO, the main objective was to study the influence of the pyrolysis process on the film properties and particularly on the NP distribution. This has not been studied before but is obviously of great importance. Up to now, the largest critical current densities were achieved when a seed layer had been deposited prior to the NP-containing film. Obviously, this prevents the NPs from agglomerating at the substrate interface during the pyrolysis^37,39^, which would disturb a controlled *c*-axis formation during the crystallisation. Thus, the seed layer preserves the epitaxial growth of the *RE*BCO phase but requires an extra deposition step. By studying the conditions that increase the precipitation of the NPs during the pyrolysis, it has been possible to design an appropriate thermal process that allows avoiding the use of a seed layer.

## Sample preparation and characterization techniques

### Synthesis of the *RE*BCO precursor solutions

The preparation of the TFA solutions employed in this work followed the procedure established by Erbe et al*.*^26,34^. In summary, the precursor salts of Gd, Ba and Cu (acetates; purity > 99.99%, Alfa Aesar) were mixed in the stoichiometric ratio 1:2:3 in deionized water and triflouroacetic acid (TFAH, 99.5+%, Alfa Aesar). Then, the mixture was dried with a rotary evaporator, and the remaining solid residue was re-dissolved in methanol. The final volume of methanol was adjusted to a gadolinium concentration of 0.25 mol/l. The Hf-containing solutions for preparing the in-situ nanocomposites were prepared following the strategy reported by Cayado et al*.*^9^ and contain additional precursor salts for the formation of 12 mol% BaHfO_3_ (BHO) within the GdBCO matrix*.*

### Preparation of the HfO_2_ nanoparticles and colloidal solutions

HfO_2_ nanocrystals were synthesized and purified according to Tang et al*.*^42^ and De Keukeleere et al*.*^43^ from 20 g tri-*n*-octylphosphine oxide (99%, Stem Chemical), 4 mmol HfCl_4_ (≥ 99.0%, Sigma-Aldrich) and Hf(O*i*Pr)_4_·iPrOH (99.9%, Sigma-Aldrich) at 360 °C for 2 h under argon atmosphere. The as-synthesized nanocrystals were dispersed in toluene, leading to a clear suspension of agglomerate-free HfO_2_ nanocrystals coated with hydrophobic phosphorus-containing ligands. Ligand exchange with a phosphonate-containing copolymer was executed according to Rijckaert et al*.*^44^ and the suspension redispersed in methanol yielding nanocrystals with a solvodynamic diameter of 10.7 nm. This nano-suspension was added to the GdBCO solution to obtain a HfO_2_-GdBCO colloidal solution.

### Thin film preparation and characterization

The precursor solutions were deposited on 10 × 10 mm (100)-oriented single crystals [LaAlO_3_ (LAO) for pristine films and SrTiO_3_ (STO) for nanocomposites] by spin coating at 6000 rpm for 30 s. The subsequent pyrolysis and growth processes are defined later in detail.

Phase purity and microstructure of the films were studied by X-ray diffraction (XRD) using a *Bruker* D8 diffractometer with Cu Kα radiation. The microstructure of the GdBCO films was further analysed by scanning transmission electron microscopy (STEM) via a *C*_s_-corrected JEOL JEM 2200-FS instrument operated at 200 kV with bright field (BF) detector. Cross-sectional STEM lamellae were prepared via the Focused Ion Beam (FIB) technique in an FEI Nova 600 Nanolab Dual Beam FIB scanning electron microscope^40^. The lamellae were extracted with the in-situ lift-out procedure with an Omniprobe extraction needle.

The Hf distribution across the GdBCO film was measured by XPS depth profiling on an S-probe XPS spectrometer with monochromatic Al radiation (1486.6 eV). Ar^+^ ion sputtering (4 keV, 3000 s total sputtering time) was carried-out in consecutive sputter cycles of 300 or 500 s. In each cycle, atoms were removed from the surface, and a quantitative analysis of the fresh surface was performed. The intensities of the Gd 4d, Hf 4d5/2, and Sr 3d peaks were registered after consecutive sputter steps and transformed into atomic concentrations by the software package CasaXPS (Casa Software Ltd., UK) using a Shirley background and Scoffield sensitivity factors.

Self-field *J*_c_ at 77 K, ${J}_{\mathrm{c}}^{\mathrm{sf}}$, was measured inductively with a Cryoscan (*THEVA*, 50 µV criterion). *J*_c_(*B*) (1 μV/cm criterion of transport *J*_c_), *T*_c_ (*T*_c,90_, i.e., the temperature at which the resistance is 90% of the value above the transition) and ∆*T*_c_ (defined as *T*_c,90_–*T*_c,10_) were studied on a 14-T *Quantum Design* Physical Property Measurement System (PPMS). The transport data were measured on 10–20 μm wide and 1 mm long tracks prepared by wet-chemical etching after photolithography. The values of the accommodation field, H*, defined as *J*_c_(H*) = 0.9 ${J}_{\mathrm{c}}^{\mathrm{sf}}$ and the exponent *α* (*J*_c_ ~ *B*^−*α*^) calculated in the range of 85—3000 mT were obtained from *J*_c_(*B*) data.

## Results and discussion

### Design of pyrolysis thermal profile

**Figure 1:**
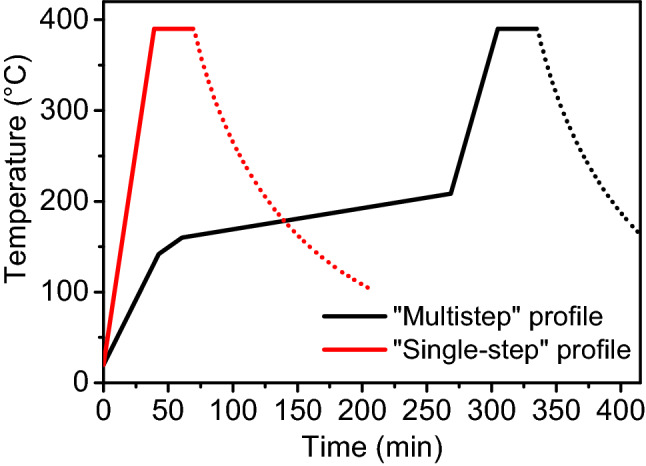
Thermal profiles of the pyrolysis employed in this study: standard “multistep” profile (black) and “single-step” profile (red). The dotted lines mark the cool-down of the furnace.

The growth of high-quality pristine GdBCO and GdBCO-BHO nanocomposite films by the in-situ approach has been previously reported by our group^9,26,34^. In those publications, the pyrolysis is carried out with a “multistep” temperature profile (Fig. 1, black curve) with a total duration of around 350 min (excluding the natural furnace cool-down). This “multistep” process was optimized for the use of TFA solutions yielding excellent results. The “single-step” profile proposed here (Fig. 1, red curve) is significantly less complex and shorter with a total duration of only 70 min depending on the maximum temperature (again excluding the natural furnace cool-down).

**Figure 2:**
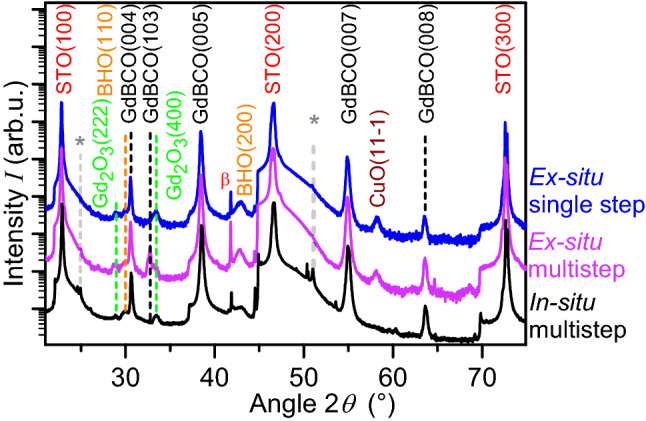
XRD diffractograms of GdBCO nanocomposites pyrolyzed with different thermal profiles. Black curve: 12 mol% BHO prepared by the in-situ approach and pyrolyzed by the “multistep” profile; colored curves: 5 mol% HfO_2_ pyrolyzed by the “multistep” profile (purple) and by the “single-step” profile (300 °C/h, blue). The reflections indicated with * come from the experimental setup, β: Cu Kβ peak of STO(200).

Since good and reproducible results had been achieved for the in-situ nanocomposites when the “multistep” pyrolysis profile had been applied in combination with the “standard” profile for the crystallisation (reported and explained in detail in Ref.^9^), the same thermal treatment was tested for solutions with preformed nanoparticles. The XRD diffractograms of the in-situ nanocomposites with 12 mol% BHO prepared with “multistep” pyrolysis show only the reflections of GdBCO(00*l*) (Fig. 2, black curve) and minor fractions of secondary phases. Consequently, these films reach ${J}_{\mathrm{c}}^{\mathrm{sf}}$ values above 7 MA/cm^2^ at 77 K. The XRD patterns of GdBCO with 5 mol% preformed HfO_2_ nanoparticles prepared by the ex-situ approach (Fig. 2, red curve) show that the same thermal treatment does not result in fully epitaxial films. The presence of a high-intensity GdBCO(103) peak proves that randomly oriented grains have nucleated leading to very low ${J}_{\mathrm{c}}^{\mathrm{sf}}$ values at 77 K of less than 0.1 MA/cm^2^. The same reduction of *J*_c_ due to the formation of randomly oriented grains in ex-situ nanocomposites has also been observed previously by Cayado et al*.* and De Keukeleere et al*.*^37,39^. This has been attributed to the fact that the NPs tend to accumulate at the substrate interface during the pyrolysis forcing the *RE*BCO phase to nucleate on top of them and so leading to the nucleation of misoriented *RE*BCO grains. The presence of BHO in the grown film indicates that the HfO_2_ NPs react with Ba during the crystallization process. This has also been observed in previous reports by Cayado et al*.* and De Keukeleere et al*.*^37,39^.

The amount of misoriented GdBCO grains in GdBCO + 5%HfO_2_ films could be successfully reduced through a “single-step” pyrolysis up to the same final temperature, 390 °C, as in the “multistep” process and with a heating ramp of 300 °C/h. The blue curve in Fig. 2 shows a clear reduction of the intensity of the GdBCO(103) peak compared to the film pyrolyzed by the “multistep” profile. Therefore, it seems possible to alter the nucleation of GdBCO by only changing the profile of the pyrolysis. If the nucleation of misoriented grains is mainly attributed to the accumulation of NPs at the interface, the “single-step” pyrolysis has obviously an effect on the NP distribution with a reduced accumulation at the interface. If the accumulation is lower, larger areas of the substrate should be free of NPs allowing GdBCO to nucleate without disturbances for the *c*-axis orientation. These results indicate that it might be possible to modify the distribution of the NPs within the GdBCO matrix in an even more systematic way and, therefore, tune the nucleation and growth of GdBCO by the choice of the pyrolysis thermal profile.

### Influence of pyrolysis parameters on the NP distribution

**Figure 3:**
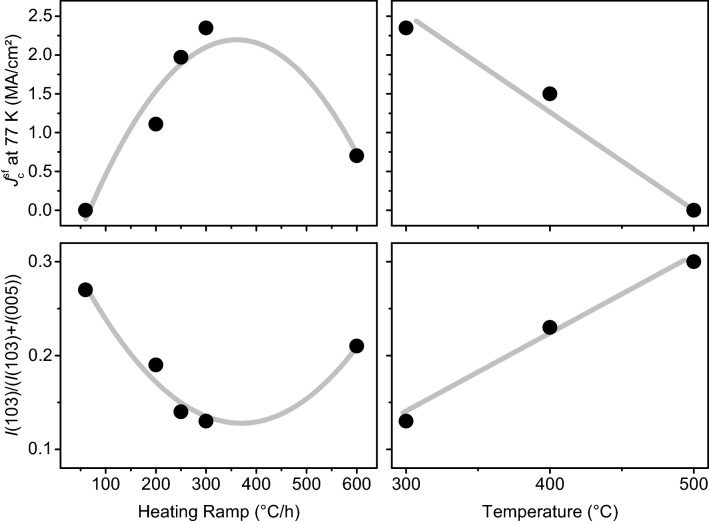
Dependency of ${J}_{\mathrm{c}}^{\mathrm{sf}}$ at 77 K (upper panels) and the GdBCO(103) peak intensity ratio calculated by Eq. (1) (lower panels) on heating ramp (left, pyrolysis temperature = 300 °C) and pyrolysis temperature (right, heating ramp = 300 °C/h), respectively, of the “single-step” pyrolysis.

Several films were pyrolyzed using a “single-step” profile with different heating ramps and maximum temperatures (pyrolysis temperature from now). Subsequently, the films were grown using the growth process reported in Ref.^9^ with a crystallisation temperature of 810 °C and an oxygen partial pressure of 50 ppm. We varied the heating ramp between 100 and 600 °C/h at constant pyrolysis temperature (300 °C) and the pyrolysis temperature between 300 and 500 °C at constant heating ramp (300 °C/h). There is a clear dependency of ${J}_{\mathrm{c}}^{\mathrm{sf}}$ at 77 K on both heating ramp and pyrolysis temperature, Fig. 3 top panels. The larger the heating ramp and the lower the pyrolysis temperature, the larger ${J}_{\mathrm{c}}^{\mathrm{sf}}$ at 77 K. Both tendencies reach an optimum, though. Heating ramps above 300 °C/h lead to the formation of cracks and buckling, which is detrimental for *J*_c_ due to obstacles that obstruct the current path and inhomogeneities that affect the effective thickness of the films. Furthermore, such defects contribute to an increase of misoriented grains^45^. A pyrolysis below 300 °C is not sufficient to burn all the organic compounds unless much larger dwell times are used which again have a negative impact on *J*_c_^46^.

The amount of misoriented grains is represented by the GdBCO(103) peak in θ–2θ scans, Fig. 2. The relative amount can be quantified by the following intensity (*I*) ratio:

1 $$\frac{{I}_{\mathrm{GdBCO}(103)}}{{I}_{\mathrm{GdBCO}(103)} + {I}_{\mathrm{GdBCO}(005)}}$$

The dependence of this ratio on heating ramp and temperature, Fig. 3 lower panels, show the opposite trends to ${J}_{\mathrm{c}}^{\mathrm{sf}}$. This means ${J}_{\mathrm{c}}^{\mathrm{sf}}$ is indeed limited by the amount of misoriented grains, which in turn is related to the distribution of the preformed nanoparticles, i.e. the amount of nanoparticles at the film-substrate interface.

**Figure 4:**
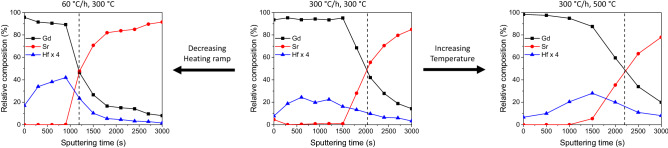
XPS depth profiles of three grown films pyrolyzed at different heating ramps and temperatures. The black dashed lines indicate the interface between film (left side) and substrate (right side), defined at the point where the Gd concentrations is reduced to half its initial value.

In order to support this hypothesis, the nanoparticle distribution in the GdBCO matrix has been further investigated. For that, XPS depth profiles were used to determine the local composition throughout the volume of the films. Similar analyses have been used before to depict the NP distribution in the films^39,40^. Figure 4 compares the XPS elemental depth profile of the optimum sample (in the middle), which had been pyrolyzed at 300 °C with a heating ramp of 300 °C/min, with a film pyrolyzed with a lower heating ramp (60 °C/min, left) and another one pyrolyzed at a higher pyrolysis temperature (500 °C, right). While for the optimum sample, the Hf signal is constant (around 5%) across the thickness of the film, a clear increase of Hf from the film surface to the substrate interface is visible in the other two films meaning that the accumulation of NPs at the interface is larger than in the optimum sample. In Fig. 4, a factor 4 has been applied to the Hf concentration for a better visualization of the NP distribution. This is in accordance with the dependencies of *J*_c_ and the amount of misoriented grains shown in Fig. 3.

**Figure 5:**
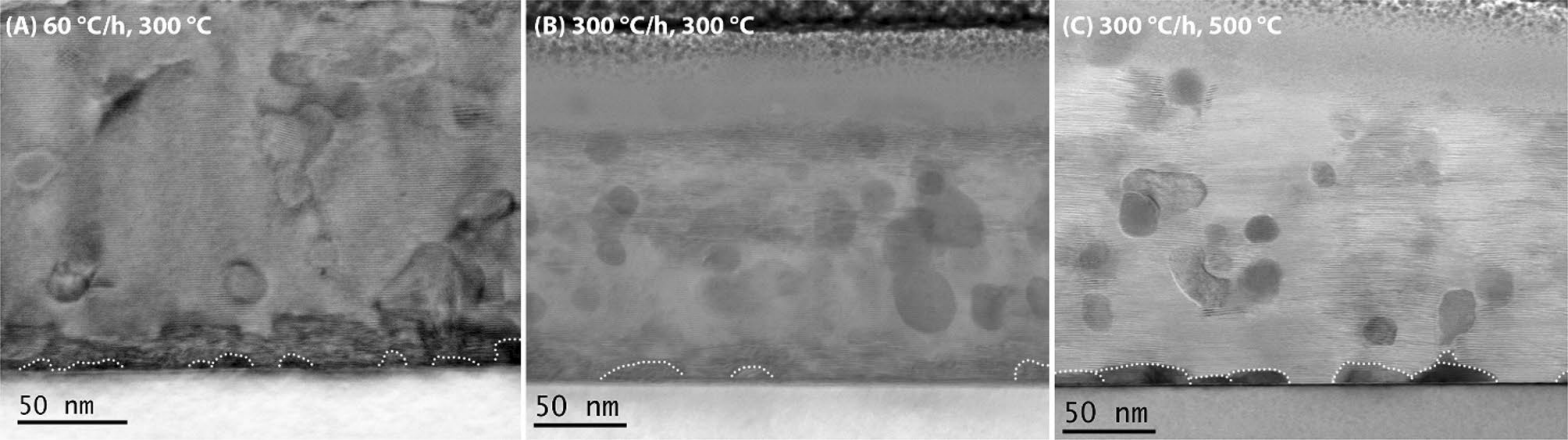
BF-STEM images of three grown films pyrolyzed with different heating rates (**A**, **B**) and at different temperatures (**B**, **C**). Clearly, a pyrolysis with (**A**) lower heating ramps or (**C**) at higher temperatures leads to a large accumulation of NPs at the interface.

The BF-STEM images in Fig. 5 serve as further confirmation for the changes in the NP distribution with varying pyrolysis conditions. Clearly, the accumulation of NPs at the interface is much more severe at 500 °C (right) or a heating ramp of 60 °C/min (left) than for the optimum sample (centre). This corresponds exactly to the XPS elemental profiles and the amount of misoriented grains shown before and, therefore, explains the differences in *J*_c_. Remarkably, the coarsening of the NPs in general, including in the matrix, is also more accentuated in the 500 °C sample, which might further decrease *J*_c_. Altogether, increasing the total time of the pyrolysis by decreasing the heating ramp or increasing the temperature seems to enhance the movement of the NPs leading to more aggregation and, finally, severe sedimentation. In return, NPs can be “frozen” in the matrix through a faster pyrolysis.

**Figure 6:**
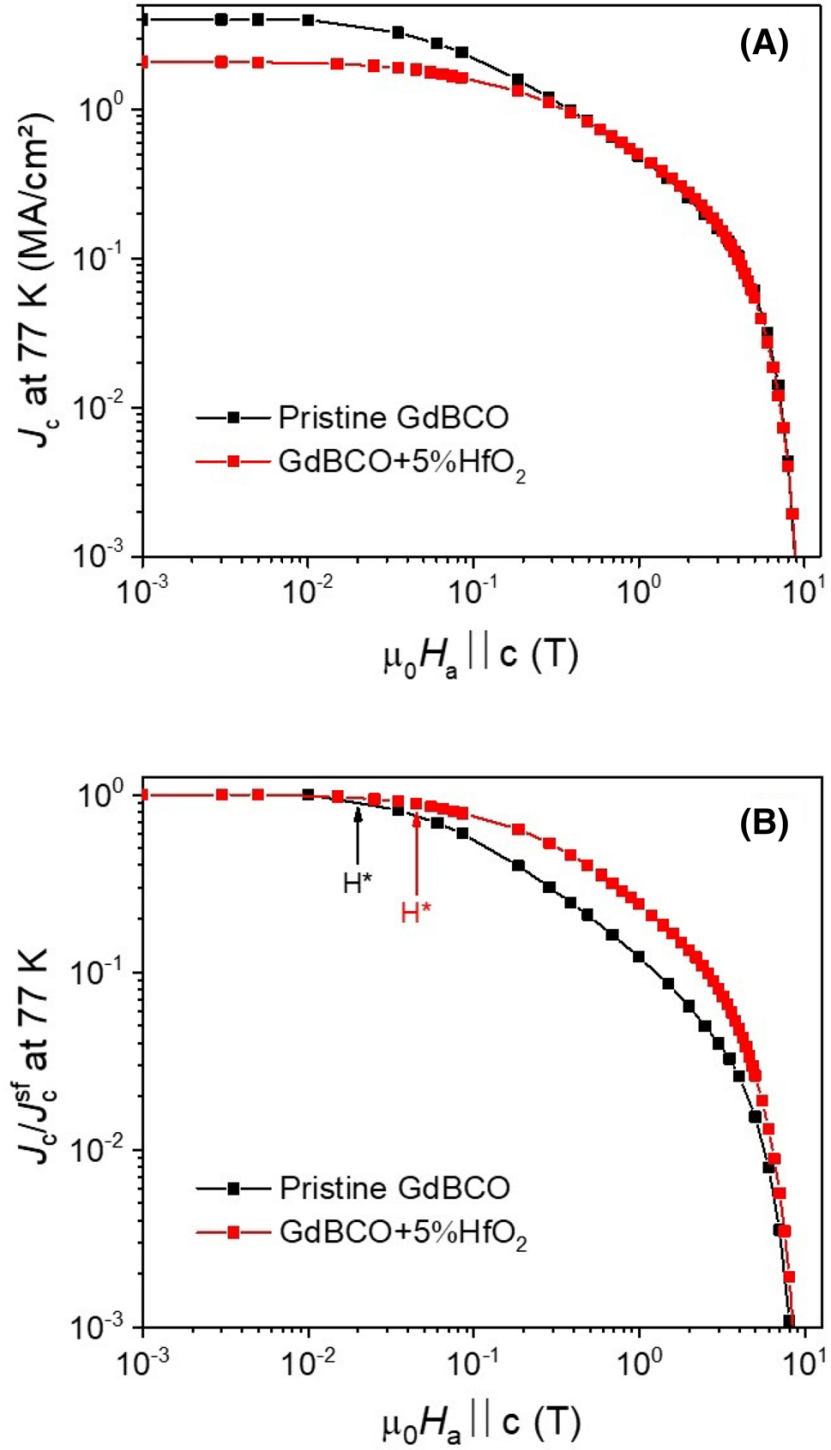
Magnetic field dependence of absolute *J*_c_ at 77 K (**a**) and normalized to *J*_c_(0) (**b**) of GdBCO + 5 mol%HfO_2_ pyrolyzed at 300 °C/h up to 300 °C (red curves) in comparison with pristine GdBCO (back curves).

All films, excluding those with zero *J*_c_ or with defects due to too fast a heating ramp during the pyrolysis, have very similar *T*_c_ values of ~ 94–94.5 K and ∆*T*_c_ ~ 1.1–1.5 K. In particular, the optimum film (300 °C/h, 300 °C) presents a *T*_c_ ~ 94.5 K with ∆*T*_c_ ~ 1.1 K The field dependencies of absolute and normalized *J*_c_ at 77 K of the optimum film (300 °C/h, 300 °C) is compared to a pristine GdBCO film pyrolyzed in the usual “multistep” process, Fig. 6. The nanocomposite films show a ${J}_{\mathrm{c}}^{\mathrm{sf}}$ value at 77 K of ~ 2.1 MA/cm^2^, which is remarkably lower than for the pristine film or the in-situ GdBCO nanocomposites. The non-perfect epitaxial growth of the GdBCO matrix in this case due to some residual accumulation of NPs at the interface is most likely the main reason for this difference, although the depletion of Ba due to the consumption by the HfO_2_ NPs during BHO formation could also play a role. However, the nanocomposite presents a smoother decay of *J*_c_ with magnetic field with a larger accommodation field H* ~ 45 mT (pristine GdBCO ~ 20 mT) as well as a lower exponent *α* ~ 0.65 (pristine GdBCO ~ 0.8). The increase in H* for the nanocomposite implies that the single vortex pinning regime is extended with respect to the pristine film.

Figure 6b clearly shows that the ex-situ approach has a great potential for increasing the performances of *RE*BCO films. A pinning improvement at medium fields is obvious but more work has to be done to tap the full potential. Even for the best pyrolysis conditions determined in this work, there is still a certain tendency of the NPs to accumulate at the interface. Thus, some misoriented grains are still present limiting the *J*_c_ values by reducing the effective cross section of the films. In order to further minimize or even completely prevent this behaviour, an even faster pyrolysis might be necessary. However, this is not possible with the full-TFA solutions employed in this study because of the appearance of cracks and buckling. A new solution formulation towards low fluorine content could be the first step for accelerating the pyrolysis and completely avoiding the accumulation of NPs at the substrate interface.

## Conclusions

This work focused on GdBCO + 5 mol% HfO_2_ to study the influence of the pyrolysis thermal profile on the final structural and superconducting properties of CSD-grown nanocomposite thin films. The crystalline quality and superconducting properties of the ~ 220 nm thick films improved for increased heating ramps and/or decreased pyrolysis temperatures. This is related to the NP distribution, which is obviously affected significantly by the pyrolysis profile: With increasing pyrolysis temperature or decreasing heating ramp, respectively with increasing total time of the thermal process, agglomeration and sedimentation of NPs at the substrate interface are enhanced. This increases the density of misoriented grains and, therefore, decreases *J*_c_. Best parameters for the pyrolysis profile were a heating ramp of 300 °C/h and 300 °C for the maximum pyrolysis temperature, which led to films with *T*_c_ values of up to 94.5 K and ${J}_{\mathrm{c}}^{\mathrm{sf}}$ of 2.1 MA/cm^2^ at 77 K. These results show the potential of the ex-situ nanoparticle formation approach. An even more homogenous distribution of the NPs may be achieved by accelerating the pyrolysis further. This, however, is not possible with the full-TFA solutions used in this work, which are limited to heating ramps of 300 °C/h.
